# Supplementary material for: Specific IgE and Basophil Activation Test by Microarray: A Promising Tool for Diagnosis of Platinum Compound Hypersensitivity Reactions
Source: Int J Mol Sci. 2024 Mar 31;25(7):3890. doi: 10.3390/ijms25073890 (PMC11011729; doi:10.3390/ijms25073890)
Supplement: Supplementary file 1 [file ijms-25-03890-s001.zip › ijms-2903678-supplementary.pdf]

Supplementary Figures

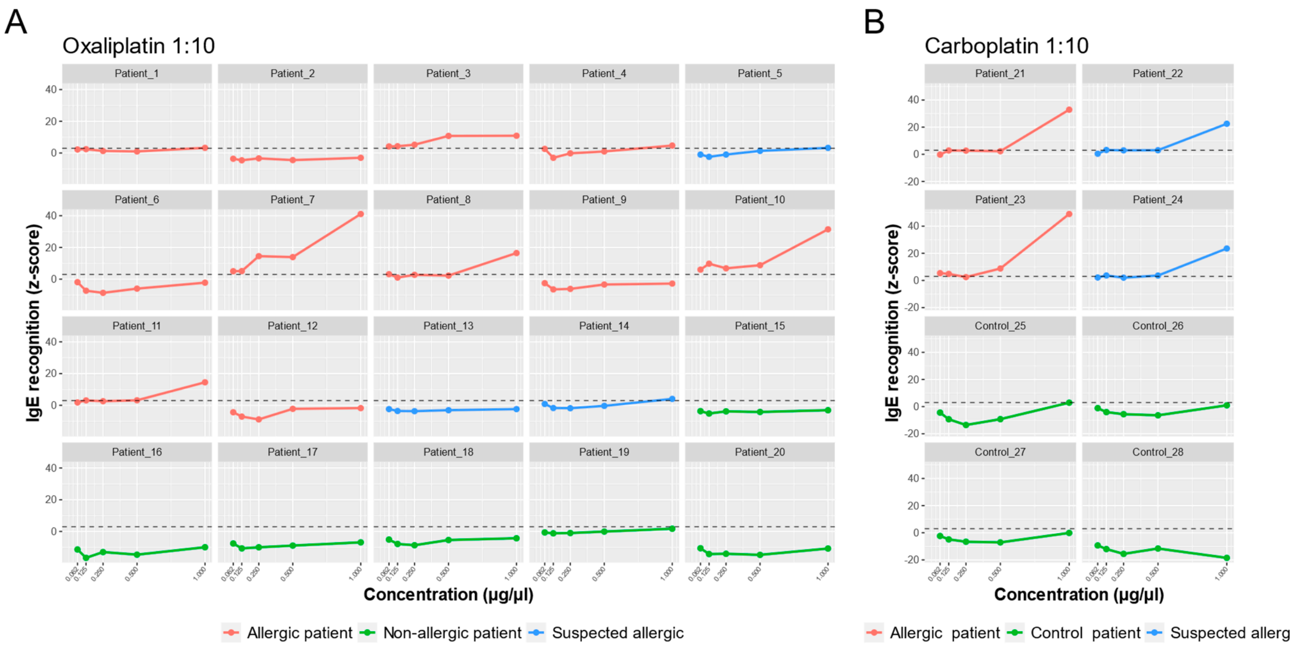

Supplementary Figure S1. sIgE microarrays immunoassay. The y-axis shows the average IgE binding represented as average Z-scores. The x-axis shows the drug concentration: oxaliplatin (A) and carboplatin (B) 1:10 molar ratio. The standardized fluorescence intensity represented as the average Z-score was considered positive if it exceeded 3 (dotted line).

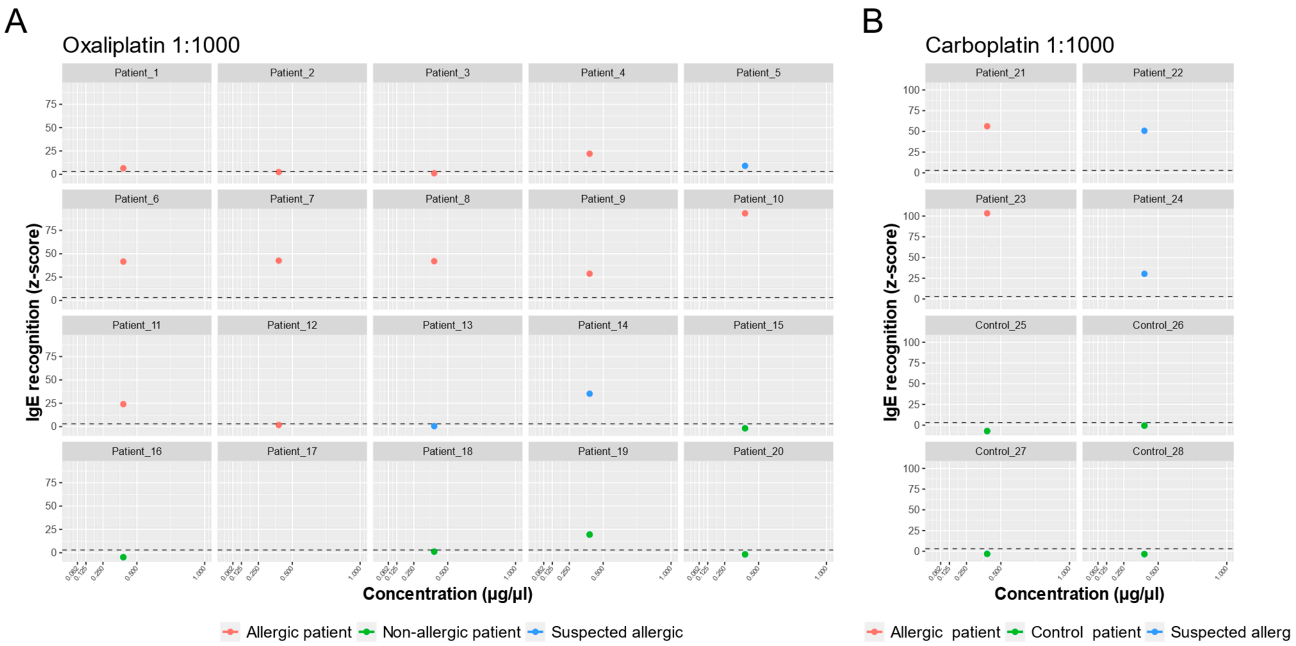

Supplementary Figure S2. sIgE microarrays immunoassay. The y-axis shows the average IgE binding represented as average Z-scores. The x-axis shows the drug concentration: oxaliplatin (A) and carboplatin (B) 1:1000 molar ratio. The standardized fluorescence intensity represented as the average Z-score was considered positive if it exceeded 3 (dotted line).

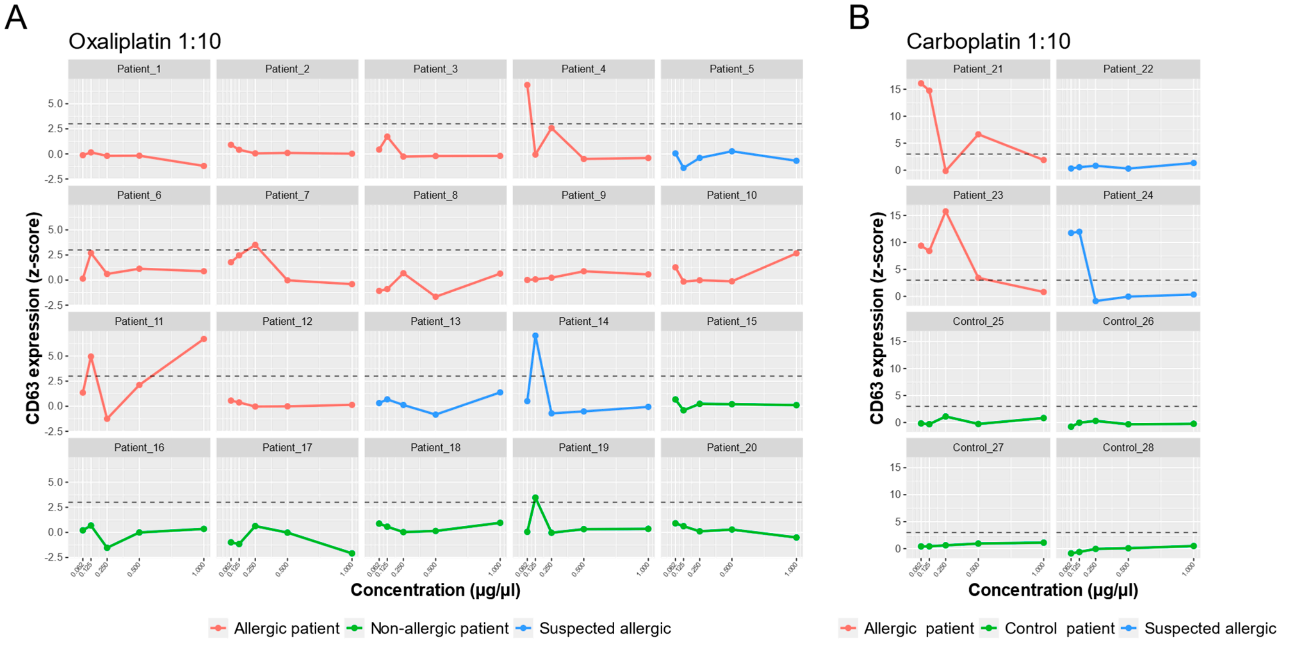

Supplementary Figure S3. BAT-microarray immunoassay. The y-axis shows the average CD63 expression represented as average Z-scores. The x-axis shows the drug concentration: oxaliplatin (A) and carboplatin (B) 1:10 molar ratio. The standardized fluorescence intensity represented as the average Z-score was considered positive if it exceeded 3 (dotted line).

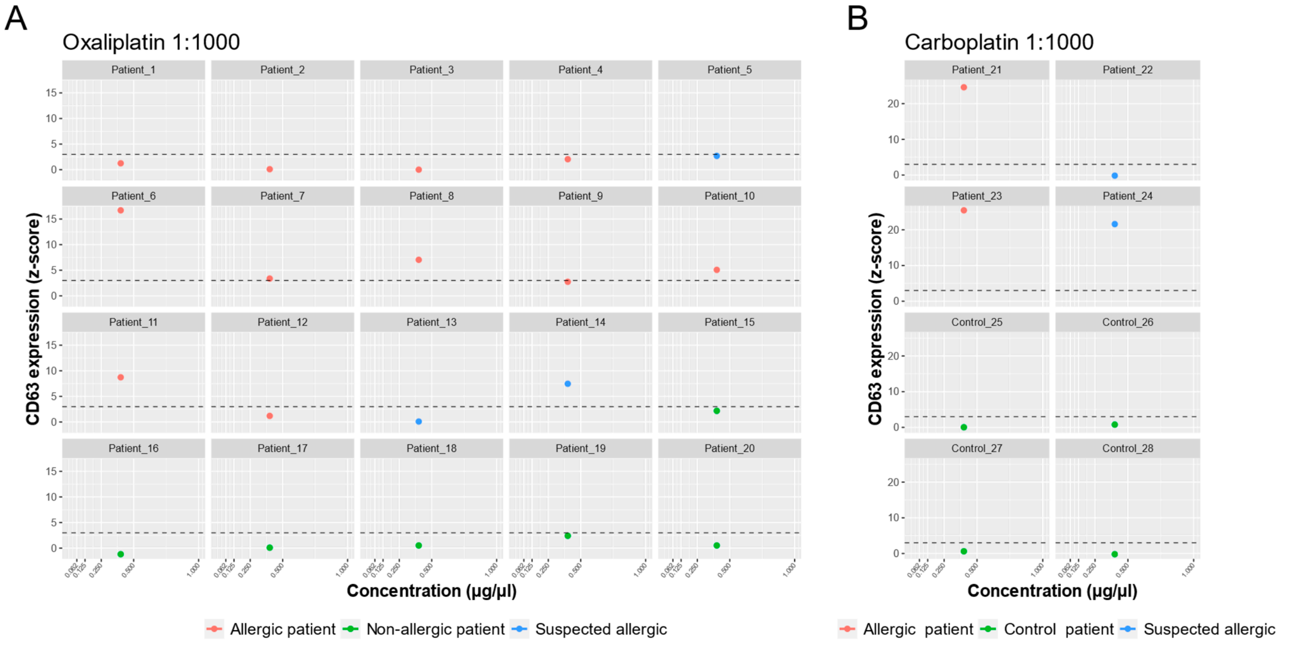

Supplementary Figure S4. BAT-microarray immunoassay. The y-axis shows the average CD63 expression represented as average Z-scores. The x-axis shows the drug concentration: oxaliplatin (A) and carboplatin (B) 1:1000 molar ratio. The standardized fluorescence intensity represented as the average Z-score was considered positive if it exceeded 3 (dotted line).
